# A qualitative exploration of the reasons and influencing factors for pregnancy termination among young women in Soweto, South Africa: a Socio-ecological perspective

**DOI:** 10.1186/s12978-024-01852-8

**Published:** 2024-07-23

**Authors:** Khuthala Mabetha, Larske M. Soepnel, Derrick SSewanyana, Catherine E. Draper, Stephen Lye, Shane A. Norris

**Affiliations:** 1 SA MRC/Wits Developmental Pathways for Health Research Unit, Department of Paediatrics, Faculty of Health Sciences, School of Clinical Medicine, University of the Witwatersrand, Private Bag X3, Wits, Johannesburg, 2050 South Africa; 2 Julius Global Health, Julius Center for Health Sciences and Primary Care, University Medical Center Utrecht, Utrecht University, Utrecht, the Netherlands; 3 Department of Physiology, Temerty Faculty of Medicine, University of Toronto, Toronto, Canada; 4 School of Health and Human Development, University of Southampton, Southampton, UK

**Keywords:** Termination of pregnancy, Socio-ecological model, Reproductive health, Women of reproductive age

## Abstract

**Background:**

Pregnancy termination is an essential component of reproductive healthcare. In Southern Africa, an estimated 23% of all pregnancies end in termination of pregnancy, against a backdrop of high rates of unintended pregnancies and unsafe pregnancy terminations, which contributes to maternal morbidity and mortality. Understanding the reasons for pregnancy termination may remain incomplete if seen in isolation of interpersonal (including family, peer, and partner), community, institutional, and public policy factors. This study therefore aimed to use a socio-ecological framework to qualitatively explore, in Soweto, South Africa, i) reasons for pregnancy termination amongst women aged 18–28 years, and ii) factors characterising the decision to terminate.

**Methods:**

In-depth interviews were conducted between February to March 2022 with ten participants of varying parity, who underwent a termination of pregnancy since being enrolled in the *Bukhali* trial, set in Soweto, South Africa. A semi-structured, in-depth interview guide, based on the socioecological domains, was used. The data was analysed using reflexive thematic analysis, and a deductive approach.

**Results:**

An application of the socio-ecological framework indicated that the direct reasons to terminate a pregnancy fell into the individual and interpersonal domains of the socioecological framework. Key reasons included financial dependence and insecurity, feeling unready to have a child (again), and a lack of support from family and partners for the participant and their pregnancy. In addition to these reasons, Factors that characterised the participants’ decision experience were identified across all socio-ecological domains and included the availability of social support and (lack of) accessibility to termination services. The COVID-19 pandemic and resultant lockdown policies also indirectly impacted participants’ decisions through detrimental changes in interpersonal support and financial situation.

**Conclusions:**

Amongst the South African women included in this study, the decision to terminate a pregnancy was made within a complex structural and social context. Insight into the reasons why women choose to terminate helps to better align legal termination services with women’s needs across multiple sectors, for example by reducing judgement within healthcare settings and improving access to social and mental health support.

**Supplementary Information:**

The online version contains supplementary material available at 10.1186/s12978-024-01852-8.

## Abstract (plain language summary)

In South Africa, where the number of unintended pregnancies is high, we need an improved understanding of the main reasons why women terminate their pregnancies and what factors characterise this decision. Aside from individual factors, this should also be seen within the context of their environment, including relationships, community, and institutions. We therefore aimed to explore women’s reasons for choosing to terminate their pregnancy through semi-structured in-depth interviews with participants. We included ten participants from Soweto, South Africa, who had undergone a pregnancy termination. The main reasons for terminating a pregnancy had to do with personal factors and reasons related to their social relationships and support. These included financial insecurity, not feeling ready to have a child (again), and lack of support from family or partners. We also found factors that characterised how the participant experienced the decision, such as barriers to getting a safe (legal) pregnancy termination. We found that amongst South African women, the decision to terminate is made in the context of their complex (social) environment. Insight into the reasons why women choose to terminate helps to better align legal termination services with women’s needs, for example by reducing judgement within healthcare settings and improving access to social and mental health support.

**Supplementary Information:**

The online version contains supplementary material available at 10.1186/s12978-024-01852-8.

## Background

The annual rate of pregnancy termination for Southern Africa is estimated at 30 per 1000 women of reproductive age, with an estimated 23% of all pregnancies ending in termination of pregnancy in Southern Africa between 2015–2019 [1]. Particularly in low- and middle-income countries, unsafe pregnancy termination remains a major cause of maternal mortality and morbidity amongst young women [2, 3]. This undermines targets to reduce maternal mortality [4] and highlights the importance of improving access to safe pregnancy termination services. This is particularly relevant against a backdrop of high prevalence of unintended pregnancies, which made up an estimated 65% of pregnancies in Southern Africa between 2015–2019 [1]. As such, access to safe services for termination of pregnancy in response to an unintended pregnancy, is key for increasing reproductive autonomy [5, 6].

Termination of pregnancy is defined as the act of ending a pregnancy through expulsion of the contents of the uterus of a pregnant woman, often through medical, surgical, or non-surgical means [7]. In South Africa, termination of pregnancy has been legal since 1996, when the Choice on Termination of Pregnancy Act was introduced, which allows for termination in the first trimester and condition-restricted provision of termination of pregnancy up to 20 weeks and is inclusive of all women of any age [8, 9]. As a result, mortality and morbidity related to pregnancy termination decreased by over 90% between 1997 and 2002 [10]. Nevertheless, a significant proportion of women still undergo unsafe, illegal termination of pregnancy in South Africa [9], defined as a termination performed outside of the Termination of Pregnancy Act, by either individuals who are not registered or trained to perform abortions, or in an environment that is not a designated health facility, or both [11, 12]. Barriers to accessing safe and legal termination services therefore remain, due to factors such as stigma, negative attitudes towards termination from healthcare providers, conscientious objection of healthcare providers, human resource challenges, fragmented health service provision, and limited access to designated facilities and services such as post-termination counselling [13, 14, 15].

An understanding of the reasons for pregnancy terminations is important to improve alignment of pregnancy termination services with women’s needs around this common but complex and stigmatized reproductive event. Past studies, including from South Africa, that focused on quantitative determinants, have found and association with socioeconomic status, maternal age, and education level, amongst other factors [8, 16–21]. However, these characterisations do not give insight on women’s own expression of their reasons to terminate a pregnancy. Qualitative explorations of the decision-making process towards termination, while scarcer, seem to align on the complexity of women’s decisions to terminate a pregnancy [22–24].

Reasons provided by women have included those that are more ‘practical’ (financial reasons, schooling opportunities), problems with pregnancy timing, and more existential reasons, such as not being ready to be a mother or stigma around pregnancy [22–26]. In addition, existing evidence has pointed towards the importance of a social network in making the decision to terminate, with a particular focus on the partner [24, 27–29]. For example, a South African study that explored the reasons for only legal pregnancy terminations, identified “problems with partners” as one of the overarching reasons for choosing to terminate a pregnancy [23]. One study conducted in Kenya found that male partners were the main decision-makers around terminating pregnancies [28], emphasizing the need to explore women’s agency in choosing for termination. In an urban South African context with persisting socioeconomic inequalities, young women face many social challenges, including economic challenges, social and familial stressors and conflict, environmental safety problems [30], and high prevalence of gender-based violence [31]. Therefore, in this setting in particular, our understanding of the reasons to terminate (both legally and illegally) may remain incomplete if seen in isolation of interpersonal (including family, peer, and partner), community, institutional, and public policy factors. In addition, the COVID-19 pandemic, and the strain it placed on local and national resources [32, 33], was found to heighten restrictions on sexual and reproductive health, including access to safe pregnancy termination services in Africa [34] and South Africa [35]. The potential role of the COVID-19 pandemic, with its myriad of social, medical, and economic consequences, therefore, also requires consideration.

This study aimed to qualitatively explore, within a socioecological framework, i) young women’s reasons for terminating a pregnancy and ii) factors characterising how women make and experience the decision to terminate.

## Methods

### Study design and conceptual framework

**Fig. 1:**
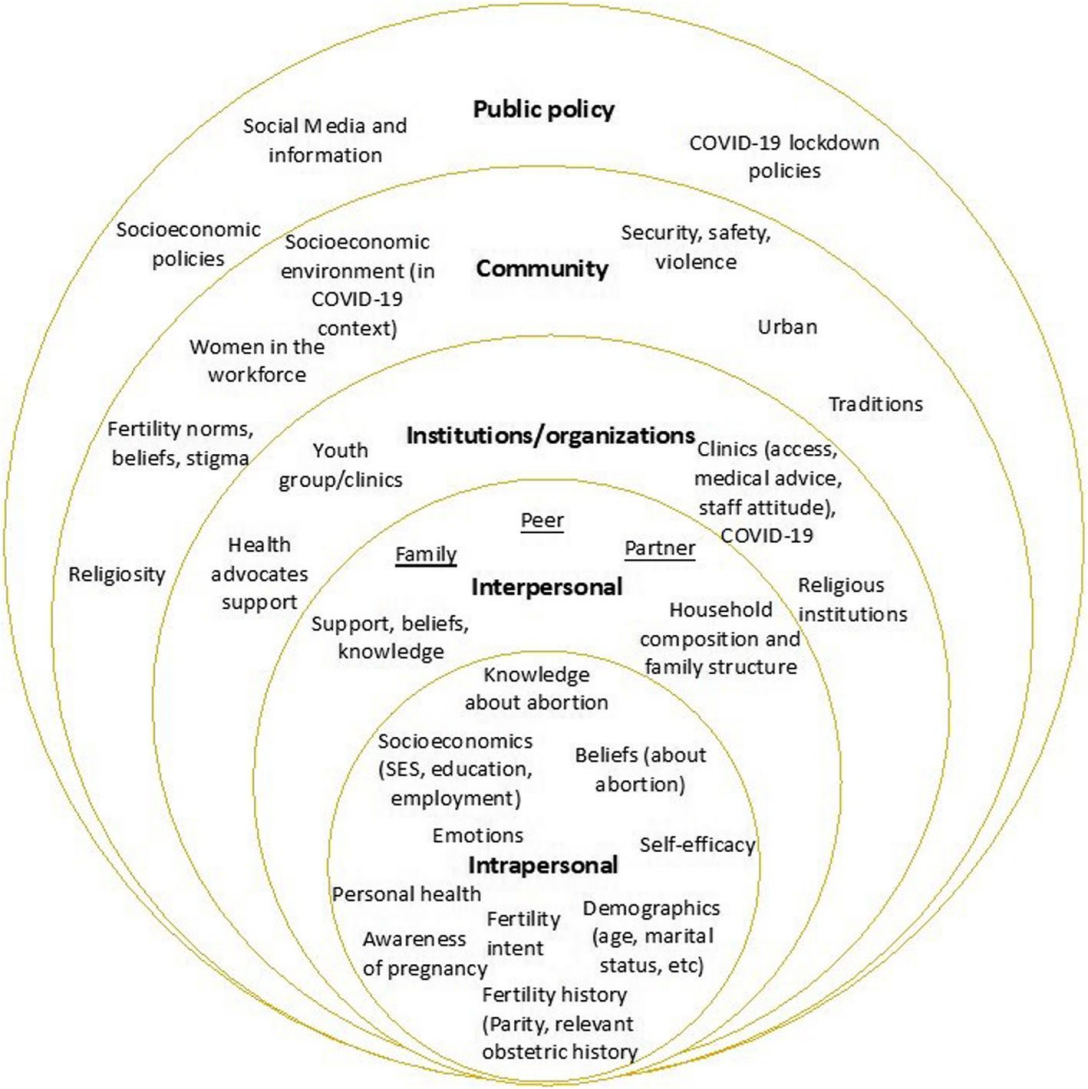
Conceptual framework for the study, adapted from the socioecological model

This study employed a deductive qualitative approach, applying a socioecological model framework [36] in order to gain a meaningful contextual understanding of the reasons why young women terminated their pregnancies. The socioecological model considers the multifaceted levels within a society and how individuals and the environment interact within a system. The framework posits that public health and health behaviors both affect and are affected by various contexts within individual, interpersonal, organizational, community, and policy factors (the five domains). Based on this model, and integrating existing evidence about the reasons for termination, discussed under “Background”, the authors developed a conceptual framework show in Fig. 1, which informed the methodological approach of this study, from data collection to analysis.

### Setting

This study was nested in the Healthy Life Trajectories Initiative (HeLTI), and specifically the *Bukhali* randomised control trial, which examines the effects of a complex intervention aimed at optimising the health of young women (18–28 years) in preconception, during pregnancy, and postnatally in Soweto, South Africa [37]. In terms of family and household dynamics, qualitative exploration of young women’s household environments found that young Black women in Soweto experience significant material and relational difficulties within their households, and these were found to negatively affect young women’s emotional well-being [38]. The impact of gender inequality and gendered roles on young women’s childbearing has also been previously characterised [39–41]. Marriage rates are declining in South Africa, and are lower amongst young black women, likely due to a combination of cultural, historical, and economic factors [42]. For example, findings from 2008 showed that only 39% of black South African mothers were ever-married [42]; more recent research from Soweto amongst 18–28-year-old women has similarly found low marriage rates [43, 44]. In Gauteng, the province where Soweto is located, the unemployment rate in the first months of 2023 was 34.3%, and unemployment was highest amongst youth aged 15–34 years old [45]. Within this setting, a previous study in Soweto reported a lifetime prevalence of termination of pregnancy of 15.3% [46]. In *Bukhali*, termination of pregnancy was observed to occur in about 5.2% of pregnancies enrolled prior to 20 weeks’ gestational age.

### Data collection

The total number of all women enrolled in the intervention and control arms of the trial was 6,800. In the current study, all women in the intervention and control arms of *Bukhali* who had undergone a termination of pregnancy since their enrolment in the trial (prior to January 2022) were invited to participate in an in-depth interview (*n* = 21). The approximate length of time between the pregnancy termination and the interview was between 4 and 12 months. Ten participants agreed to be interviewed and were included in the study. Reasons for not participating (*n* = 11) included being out of town, not being contactable after two attempts, and not being interested in being interviewed about termination in pregnancy. Some participants did not attend a booked appointment but did not provide a reason for not attending. After the 6th interview, participants interviews were providing fewer new insights, and by the 10th participant we reached data saturation. Participants who experienced a pregnancy loss (spontaneous abortion) were not included in this study.

Individual, semi-structured in-depth interviews were conducted with the participants during February to March 2022 at the trial’s research centre within the precinct of Chris Hani Baragwanath Academic Hospital in Soweto. Interview guide questions were semi-structured and were developed by the study team and used for prompting where necessary (Additional file 1). The interview questions were developed according to the domains of the socioecological framework, namely: individual-level factors, interpersonal factors, institutions/organisations or community-level factors, public policy, and media. Additional questions around COVID-19 were also included, given the timing of the study during the pandemic.

The interviews were then conducted face-to-face by KM, and a notetaker (LS), using both English and other South African vernacular languages (IsiZulu, Xhosa and Sesotho) for participants who preferred to be interviewed in their home language. A funnel approach to interviewing was used by first building rapport with the participants, which assisted in creating a safe space where participants could speak candidly about their experiences. Rapport was built by welcoming participants and having a brief, casual conversation with them, introducing the interviewers and the interview process (including the anonymity of the data), and by starting the interview by asking a number of simple questions about the participants and their household (such as their age and who they live with). Following COVID-19 safety protocols, interviews were conducted in a quiet room that offered maximum privacy. Interviews were audio-recorded and lasted between 45–90 min. Prior to analysis, the audio recordings of the participants were transcribed verbatim and translated into English where necessary by a third-party provider.

### Data analysis

A reflexive thematic analysis approach was used to analyse the data, recognising the subjectivity of the study team in interpreting participants’ experiences [47, 48].Starting from a deductive approach, the socioecological model served as a lens through which the analysis was conducted, with each level of the model (individual, interpersonal, *et cetera*) considered an a priori domain under which our findings were organised. However, the development of themes within these broad domains employed a combined deductive and inductive approach, responding to the data at hand. This allowed flexibility to identify and interpret participants’ reasons for termination and their decision-making experiences, within each level of the socioecological model. In the first step of analysis, KM and LS familiarised themselves with the data, listening to recordings, reading transcripts, and mapping initial connections, codes, and patterns across the interviews. Thereafter, a comprehensive process of data coding was undertaken, with codes evolving throughout the process, through discussion between KM, LS, CD, and DS. Coding was done using MAXQDA Software [49]. Next, themes were developed iteratively and organised according to the relevant socioecological domain. Themes were divided into (i) direct reasons for deciding to terminate, and (ii) factors characterising how the participant experienced their decision to terminate. For each theme, exemplifying quotations from the transcripts were identified and are presented below.

### Ethical statement

This study was approved by the Human Research Ethics Committee (Medical) at the University of the Witwatersrand (M190449). All participants gave written and verbal informed consent to be interviewed, and for the interviews to be recorded, before commencing data collection. The assistance of a professional nurse was available to provide counselling to participants in the event of distress during the interview, and all participants were provided with information for relevant helpline services.

## Results

An overview of participant characteristics is shown in Table 1. Of the 20 participants, 7 were between 20–23 years old. In addition, most of the participants (6had a secondary school qualification, and almost all [9] were unemployed at the time of the interview. An overview of the themes, per socioecological domain, is shown in Table 2. Sect. “Reasons for termination of pregnancy”of the results presents themes for direct reasons for termination, which fell into the individual-level or interpersonal domains. Sect. “Factors characterising the decision to terminate” presents themes around factors characterising the decision experience, which fell into each of the 5 socioecological domains (Table 2).

**Table 1:** Overview of participant characteristics

**Variable**	Category	N (%)
**Age**	20–21	3 (30)
	22–23	4 (40)
	24–25	1 (10)
	26–27	2 (20)
**Highest level of education**	Grade 11	3 (30)
	Secondary school qualification	6 (60)
	Tertiary qualification	1 (10)
**Employment status**	Unemployed	9 (90)
	Employed	1 (10)
**Lives with (in household)**	Partner and children	1 (10)
	Parent, siblings, and/or child	8 (10)
	Other relatives	1 (10)
**Relationship status**	Single	2 (20)
	In a relationship	8 (80)
**Total number of pregnancies**	1	2 (20)
	2	6 (60)
	3	1 (10)
	4	1 (10)
**Number of terminated pregnancies, (including termination of pregnancy discussed in interview)**	1	9 (90)
	2	1 (10)

**Table 2:** Themes focusing on young women’s reasons and factors characterising the decision to terminate a pregnancy

**Domains**	**Themes**	
	**3.1. Reasons for pregnancy termination**	**3.2. Factors characterising the decision experience**
**Individual-level**	- Financial insecurity and dependence	- Sense of agency over termination
	- Lack of readiness and desire for pregnancy and motherhood	- Personal beliefs around termination
	- Impact on employment and educational opportunities	
	Pregnancy related mental and physical conditions	
**Interpersonal**	- Lack of support or stability from partner	- Social support from confidants
	- Lack of family support	- Role of partner in decision to terminate
	- Threat of an adverse impact on family dynamics	
**Institutions and organisations**		- Organisations and institutions that offer support
		- Accessibility of termination at government clinics, private clinics, and illegal termination services
**Community**		- Community socio-cultural beliefs and norms around termination and pregnancy
		- Pregnancy termination norms
**Media and policy**		- Social media portrayal and support around termination
		- Indirect impact of COVID-19 pandemic

### Reasons for termination of pregnancy

#### Individual-level themes

##### Financial insecurity and dependence

One of the main reasons for deciding to terminate their pregnancy reported by participants was financial insecurity and dependence and therefore feeling unable to adequately provide for a child.

*“We are dependent on social grant money so, bringing a baby was not an option because there are times where we would sleep without a meal. I didn’t want to bring a child into that world.” (In-depth interview [IDI] 6)*

*“What was I going to provide the child with? I know that the father of the child was going to help me, but I think now that I am this age [late 20s], I have a need for independence. So, what if he doesn’t want to contribute anything this month, then what am I going to do?” (IDI 5)*

*“I don’t have matric, I am not working, I am depending on my mother.” (IDI 3)*

One aspect of the financial pressure described by some of these participants was already having a child or children to support, and feeling unable to support another child, suggesting that the financial pressure was amplified for women who were already mothers.

*“Things are not easy for my first two kids… the load will be too great with three kids, especially being unemployed.” (IDI 2)*

Lastly, although most participants did not report a direct impact of COVID-19 on their decision to terminate, many reported changes in socioeconomic circumstances and employment, thus implying that this may have contributed to their termination decision.

*“Since COVID started, people have lost their jobs, okay, first, they were losing their jobs, but now, it is worse, because the company is following certain rules, there are things that they have to do, so they can’t hire people, so if I am not hired, I am not going to get a job, if I don’t get a job, what are my kids going to eat, so that influenced as well, yes.” (IDI 5)*

*“I was not working right, and my mother had lost her job as well [due to the COVID-19 lockdown regulations], and then I was like okay, if I have a child and my mother does not work, I am not working and the father’s family does not want the child, so things were not going to go well.” (IDI 3)*

##### Lack of readiness and desire for pregnancy and motherhood

A number of participants expressed not wanting any (more) children.

*“I don’t want them [children in the future] …The thing is I played a motherly role to my siblings as a young kid, so I don’t think I want to go back there.” (IDI 6)*

*“No, I’m okay with the children that I have. Even if I become financially stable, I’m alright.” (IDI 2)*

In addition, some participants described wanting children in the future but choosing to terminate because they did not feel psychologically prepared to become a mother or to have another child at the time of their pregnancy.

*“Because I wasn’t ready for that child… I think also because I have a small baby, so I felt that she needs more attention, because she is one-year old, so I felt that a baby plus a young child, I don’t think I will be able to balance that.” (IDI 5)*

One element of this lack of readiness was the unplanned nature of the pregnancy, with some being the result of failed contraception or not having used birth control methods. Barriers reported by participants to using contraception included negative beliefs about side effects, judgement from clinic staff when getting contraception, lack of stock, and distance to clinics. Participants described feelings of shock, unhappiness, and disappointment upon finding out they were pregnant.

*“I was so disappointed because I told myself that after this first pregnancy, I was not going to be pregnant any time soon. So, when I found out I was pregnant, I was devastated, and I had mixed emotions… I just decided that it’s the best choice for me to do to terminate until I’m financially, emotionally, and mentally ready for a second child.” (IDI 1)*

##### Impact on employment and educational opportunities

Another direct reason for some participants to terminate their pregnancy was the negative impact of the pregnancy, and subsequent childcare duties, on employment and schooling opportunities.

*“I told him [partner] that I wasn’t ready because I wanted to go to school, and that pregnancy would delay me.” (IDI 6)*

*“What if I get a job, then what am I going to do?” (IDI 5)*

##### Pregnancy-related mental and physical conditions

Two of the interviewed participants indicated that the pregnancy made them feel unwell, in terms of physical or mental health, and that this contributed to their decision to terminate. One participant in particular described experiencing self-described symptoms of depression, which she recognised from previous pregnancies, which was compounded by a lack of support from the participant’s partner (see Sect. "Interpersonal-level themes”).

*“I then started feeling sick because of the pregnancy. Pregnancy has never been good to me. I started losing weight. I had thoughts of terminating and then there was this one weekend where I felt depressed, I didn’t want my kids or anyone near me. I wanted to vanish and didn’t want anyone near me. I lost my appetite, and I would go for 3 days without water and food. I didn’t want anyone around me, I felt like I was cursed and depressed…My partner just went out and when he got back, he argued and asked, why didn’t I clean the house and said I’m always sleeping and acting like it is my first pregnancy…I then decided that first thing on Monday, I am terminating.” (IDI 2)*

*“I was sick. I was not feeling well, and I couldn’t stand for more than 2 minutes. I would need to sit down or else I would be dizzy. I wasn’t supposed to go for more than 2 hours without eating.” (IDI 6)*

#### Interpersonal-level themes

##### Lack of support or stability from partner

While some participants reported receiving support from their partners during and after the pregnancy termination, other participants were not in a stable relationship, had partners who expressed unwillingness to take responsibility for the pregnancy, or experienced negative changes to their relationship following the disclosure of their pregnancy. The lack of partner support seemed to make it more difficult for participants to accept their pregnancies and to trust in their partners, which was a reason for participants to choose to terminate their pregnancies.

“*My relationship with my partner is not that great. Having another child wouldn’t have been good.*” (IDI 2)

*“At first he was a good guy, you know how boys are, they will promise you heaven and earth, then you see that it’s okay, then things changed after I told him I was pregnant, then he started avoiding my calls, never visit me like often, …he said he was not ready to be a father, then I tried explaining, like, the circumstances at home, and what will happen to me if I do termination and all that, then he was like, he was not interested in that.” (IDI 3)*

*“Before I fell pregnant, it was alright, but then when I fell pregnant, he stopped caring and he was busy with the other girls. When I tell him about the pregnancy, he would switch the topic and we would talk about something else, so I saw that it is useless to keep talking about it, so I just took a decision to get it done.” (IDI 4)*

One participant’s partner was also in a serious relationship with another woman, and wanted to raise their child in secret, which was not acceptable to the participant.

“*I realised that this is not the kind of a partner I want to keep, you understand. And it’s clear that my child will be a secret, or he can start ghosting me anytime, as if, you know. Then I decided that I am going to terminate.*” (*IDI 8)*.

In contrast, one participant reported that, although willing and supportive, her partner was unable to financially support the child.

“*He said he is not ready. He is not financially stable, and he is young. He said he won’t be able to support the child fully. With him being young as well, he needs to sort out his household, send money to his family and take care of his siblings.” (IDI 6).*

##### Lack of support from family

A lack of support, whether emotional or practical, from family members was reported by participants as another reason to terminate their pregnancy. This included poor social relations with their families, family environments characterised by conflict and hostility, a fear of judgement, and poor communication with little to no emotional support. These circumstances often resulted in participants not disclosing their pregnancy to family members, which appeared to isolate them in their decision.

“*My mom and I don’t have a bond at all… So that’s one reason why I didn’t want to have another baby. If my mother and I were tight, I would never have gotten an abortion* [termination]*. You see if we had that relationship, she would have said, the second one now, and then she gets angry, because she is a parent. And then she says we are going to see what we can do, because my mother and I are friends, and I would have said I have my mom, we will see what we can do. So, I don’t have that with my mom, I don’t have that, that I know that my mom will be behind me, I can never count on my mom, you see.*” (IDI 8)

“*My relationship with my family is okay but I don’t tell them some of the things. I feel as if, more especially my sister, and that is why I was not able to go to her and tell her that I am like this and I plan to do this. I feel like she is too judgmental. She is the type of person who opposes everything the minute you tell her something.*” (IDI 9)

In contrast, two participants indicated that their decision to terminate was impacted by family members urging them to terminate given their families’ living circumstances and poor financial standing.

“*My mother influenced my decision to terminate. You know how mothers are, like where is your child going to stay, you can see that we are in a little house, you see, things like that. She showed me her financial situation and that I already have two other children.*” (IDI 9)

##### The threat of adverse impact on family dynamics

Some participants reported that a reason for terminating their pregnancies was to avoid an adverse reaction from their family members, ranging from disapproval to abusive treatment. The narrative of one participant showed that disclosure of a second unintended pregnancy was met with hostility from the family’s side and ultimately, a dismantled relationship, resulting in a lack of communication about the pregnancy termination, and limited contact with the family.

“*They understood when I made the mistake the first time around so, I’m inconsiderate with the second child. I am not serious about the future, and they disowned me.*” (IDI 2)

In some cases, this threat was based on traumatic experiences during previous unintended pregnancies, which had been met with hostility and tension from family members. This had led to adverse outcomes such as not being fed or being chased out of the family home. The participants’ decision to terminate their pregnancy in these cases was thus to avoid similar experiences within their families.

“*I was pregnant when I was still at school, my family was angry at me. They were not giving me food and ever since they found out that I [was] pregnant, they then stopped talking to me. I was sleeping in my mother’s bedroom, and then my big sister chased me out of my mother’s bedroom and locked it, and she told me to sleep in the dining room, .... So, after I found out [about the most recent pregnancy], I was scared, and I thought about my first pregnancy, the fact that things changed at home when I was pregnant, so I thought that having another baby will cause the same thing that was happening, so I didn’t like to keep the pregnancy because of what happened with the first pregnancy.*” (IDI 4)

“*My eldest aunt always says that she doesn’t want another child so, if I bring another child, it will be a problem. They chased me away with my first pregnancy.*” (IDI 7)

### Factors characterising the decision to terminate

#### Individual-level themes

##### Sense of agency over reproductive health and termination

Participants reported feeling like the decision to terminate their pregnancy was solely their decision, displaying a sense of autonomy that came across in most interviews.

“I was not under pressure to terminate or not, the decision was solely mine.” (IDI 5)

*“At the end of day, the baby is in my body, and whatever is going to happen, is going to affect me more than you, …, so if I say I don’t want a baby, then I don’t want it.” (IDI 8)*

Even the participants who chose to terminate because of circumstances leaving them with few alternatives (such as financial stressors), seemed to use wording that claimed the decision as their own. The role of the partner in the decision is described in more detail in Sect. “Interpersonal-level themes”.

*“I had my own reasons for doing, so what other people thought, I didn’t want to care about that.” (IDI 9)*

*“I just decided to do what will be best for me at that particular moment.” (IDI 3)*

One participant expressed not feeling like she had power to decide over her sex life. Notably, although the participant felt that she had complete autonomy in the decision to terminate her pregnancy, this decision-making power was hindered when it came to sexual self-efficacy and negotiating sex with her partner.

*“There are times when you don’t feel like it [having sex], but you find that your partner wants it, so you just do it so that he can leave me alone. So, I don’t think [I have power] when it comes to that.” (IDI 5)*

##### Personal beliefs about termination

The majority of participants expressed personal beliefs that termination was wrong or sinful to some degree, and a number of participants reported fearing consequences of the termination, whether physical or spiritual. Many of these beliefs had a religious or cultural foundation (such as Christianity or traditional cultural rituals).

*“I feel like it will haunt you as time progresses… or maybe it would lessen the chances of having another child in future.” (IDI 2)*

*“My belief is that, well I’m not really religious but I believe that termination is not right because there is a soul that you kill. So, I’m against it but at some point, I had no choice.” (IDI 1)*

While some participants reported feelings of doubt about terminating their pregnancy, many participants also described setting such beliefs aside when making their decision to terminate their pregnancy, because it was the right thing for them at the time.

*“I went against my beliefs, for the very first time, I had to put things aside about my beliefs, about what people say and what the bible says, I just told myself that okay, if it’s a sin, we all have sins, it’s just that they are different, I will deal with it in the future. I just told myself that this is the decision that I am taking… I wanted to terminate but still even that time I knew that a child is a blessing… I am not saying it is wrong or right, people have their own reasons.” (IDI 8)*

*“No [beliefs didn’t influence decision]… I think it is something that I wanted to do.” (IDI 10)*

*“If it were for me, terminating is a sin … I didn’t consider [these beliefs] at all…I mean church is church, God is the only one that can judge you.” (IDI 6)*

In contrast, rather than believing that termination was wrong, some participants expressed beliefs around trusting and respecting individual decision-making.

*“I feel good because everyone has their own reasons. I have my own reasons why I did it [terminated the pregnancy]. Like I didn’t want to think much about it because it is something that I wanted to do, so yeah.” (IDI 9)*

*“I was told to do what I believe in, right, I have to trust myself, if I am making a decision, it does not matter what decision [to terminate] I am making, if I am comfortable with the decision, I have to stick with it.” (IDI 3)*

#### Interpersonal-level themes

##### Social support from confidants

Although many kept their pregnancy or termination secret from family members or partners, participants indicated that they received support from strategically selected trusted confidants, including friends, neighbours/landlords, or select family members, following disclosure of their pregnancies. This included tacit support helping participants obtain the relevant information pertaining to termination and accompanying them to the termination centre.

“*She [friend] was with me throughout the abortion [*termination*], she was sitting with me at the clinic. She is the one that took care of me when I wanted food. She was a good friend. She asked me what it is that I wanted, whether I wanted to keep the child or terminate. I told her that I wanted to terminate. Then she accepted and said that she will go with me to the clinic, and I should tell her if there is anything that I need.*” (IDI 6)

Confidants seemed to support participants’ decisions to terminate the pregnancy, offer non-judgemental emotional support, and provide encouragement and assurance that they would be with them every step of the way.

“*My friend’s mom supported me. She also did it before, so she told me about what happens when you get there. Her words and the way she spoke to me were so encouraging.” (IDI 10)*

While such support was not reported to impact the outcome of participants’ decisions to terminate, and in fact tended to emphasise that the decision was up to the participant, it did seem to ease their decision-making experience leading up to the termination, and was perceived as unconditional of the decision made.

“*I spoke to my landlord. She said it will be okay and she encouraged me to carry on looking for work and hopefully something will give. When I decided that I wanted to terminate, she did say that the choice is mine and she referred me to someone at [clinic name] clinic. She did that by saying it is my decision and she wouldn’t block me, and she doesn’t condone it, but she is there for me.*” (IDI 2)

##### Role of partner in decision to terminate

The partner’s role in the decision to terminate, and the reason behind their (lack of) involvement varied widely between participants. Partners of some participants were actively involved in the termination decision-making, making the decision to terminate a joint decision.

“*I told him that I wasn’t ready…He also agreed as he is not ready as well. He is also not financially stable, and he is still young. He put all the cards on the table and showed me disadvantages of keeping the baby and the advantages. He did say that any decision that I made; he will support me, but I should also know that he won’t be able to support the child fully. We agreed that we will terminate and then yeah.*” (IDI 6)

“*It was a joint decision, but he was supporting me in anything. Like I said that he said I should do whatever I want. So, he was supporting me on the decision that I took.*” (IDI 5).

Contrary to these findings, other participants indicated that although they informed their partners of the pregnancy, the decision to terminate was made by the participants alone, with partners indicating they would support whichever decision. This seems to indicate that, although some participants reported feeling supported, the partners were able to distance themselves from the decision-making process.

“*He did not fight over the termination issue, and I feel like he also wanted me to terminate from the beginning, but he didn’t want to tell me or take that decision for me. He wanted me to be the one saying that.*” (IDI 8)

In contrast, some participants made the decision alone because they chose not to tell their partners about the pregnancy, or in some cases, about their decision to terminate, claiming instead that they had had a spontaneous abortion (miscarriage).

*“He did not know. I went to the doctor and the doctor scanned me and he told me how many months I have, then he gave me those pills, like he would be with me when I take them, and then when they were working. I saw the doctor and he checked me, cleaned me, and scanned me and found that I have nothing, with the pills, and then when I went to them, when I went to his mother, I told them that I was pregnant, and I got a miscarriage.*” (IDI 9)

“*Remember he was happy when I was pregnant and then I aborted but he isn’t aware that I terminated. I told him that I miscarried.*” (IDI 2)

In addition, in some cases the decision was made by the participant alone because their partner showed no interest in or took no responsibility for the pregnancy.

“*No, I told him after, that I did termination, because he had already told me, do what is best for you, if I get an abortion [*termination]*, that is none of his business.*” (IDI 3)

Lastly, one participant reported feeling pressured by her partner to terminate the pregnancy.

*“Yes, I think the pressure that I was put under…He texted me daily reminding me that I should do it [terminate the pregnancy].” (IDI 10).*

#### Institutions and organisations

##### Organisations and institutions that offer support

While many participants reported receiving limited mental health support from institutions such as clinics, there were incidental reports of organisations and institutions that were supportive of participants’ decisions to terminate. For example, in contrast to some other participants, one participant reported receiving support from clinic staff.

*“They didn’t judge me [at the local clinic] …The person [nurse] that I got there was elderly, so when she was talking to me, she was talking to me properly, and she was saying that I must not feel guilty…It helped me a lot, because I knew that I was not the only one.” (IDI 5)*

For another participant, support from organisations included a youth organisation and church.

*“It was [organisation], It is a non-profitable…It’s an organization that helps kids, like it has different categories, the one [category] that I joined is “Decision”, like you will find that there are people who had abortions* [terminations]* before and the ones that want to do it, do you understand, yeah, to me they helped.” (IDI 3)*

*“I could say church… they do not support termination, but they support whatever decision you take, stick to it, it doesn’t matter, what decision, but as long as you understand it, it is okay.” (IDI 3)*

##### Accessibility of termination at government clinics, private clinics, and illegal termination services

Participants attended either government facilities, illegal termination services, or private clinics for their termination. A number of participants stated that private clinics were too expensive for them to use. At government clinics, participants related mixed experiences with accessibility in terms of reachability and staff attitudes. While some participants had a positive experience with clinic staff (“They were okay… the way they spoke to me.”- IDI 10), others reported feeling judged or poorly treated.

*“With hearing from people that the nurses are harsh and rude while people are watching, they don’t understand why you want to terminate.” (IDI 7)*

*“She [nurse] had a big attitude…but I told myself, like I want to do it, like I had made up my mind that I want this to happen.” (IDI 9)*

In addition, multiple participants described that clinic staff booked an appointment to terminate their pregnancy on a date too far along in their pregnancy.

*“I went to [name termination clinic] and when I got there, they booked another date for me, and by that time I would have been 7 months, so I talked to a friend who knows a friend and a friend, so I got the number of this lady and we met, and she gave me the pills and that was it… Yes, cause the private is also very expensive and they charge based on your months of pregnancy, you see…” (IDI 5).*

Since termination of pregnancy is not a service delivered at all local clinics, difficulty traveling to the correct clinic was another barrier to accessing termination services.

*“For preventing, that clinic is close. But the termination clinic is far, and I have to catch two taxis to get to that clinic.” (IDI 2)*

As a result of such barriers, in some cases, participants saw illegal termination services as the most accessible option. While such illegal services were largely experienced as judgement-free, one participant did report feeling that the procedure might be unsafe and risky. In addition, at least one participant held the perception and fear that the procedure would have an adverse impact on her future fertility.

*“What if I bleed too much and faint or something, and I was just with my kids, you know, things like that. So, it was risky and that was what I was scared of more than anything. That what if something happens, there will be no one to help me.” (IDI 5)*

Some participants reported that the COVID-19 pandemic had a negative impact on the accessibility at clinics due to the impact of the pandemic on the healthcare sector.

*“Because of COVID-19, the clinics don’t take in more than 10 people so I was lucky as I was tenth on the list. I would have to be turned back if I arrived a minute later and I would have then had to create another story for my partner.” (IDI 4)*

*“Due to COVID-19, over there you don’t get doctors, because they told me to turn back when I told them what I was there to do, then someone told me about someone that they know, then I went there, that is where I did it.” (IDI 9)*

#### Community

##### Community socio-cultural beliefs and norms around pregnancy and termination

Participants indicated that their churches, family, and broader community hold certain cultural beliefs and norms around both pregnancy and termination. Many participants reported that their communities see children as a blessing and termination of pregnancy as unacceptable.

“*Their belief is, they believe that I am a girl and that a child is a blessing.*” (IDI 9).

“*In my church, you must not terminate at all*…*My mother wouldn’t have allowed it. She would have said that I bring the child to the world, we would rather suffer with the child instead of killing an innocent soul.*” (IDI 6)

Although these were commonly reported beliefs, which seem to influence participants’ own beliefs around termination (see Sect. “Individual-level themes”), the majority of participants held that it did not influence or deter them from their decision to terminate their pregnancy. Nevertheless, participants expressed the presence of stigma and judgement around termination in their community, and that these judgements tend to be based on the beliefs described above, that termination is unacceptable.

“*The only concept they have is that it [termination] is bad, you don’t kill. You can’t engage in something and when the results come, you decide to terminate. The community doesn’t take termination so well. There is judgement about it.*” (IDI 3)

Participants reported experiencing stigma and judgement from their community, including perceived gossip or through direct comments received by participants.

“*Once they [people in their community] know that your terminated, they are going to look at you in a different way, you see, like this one terminated, like there are always going to be words around your termination, even if they normalize termination, but we always come across people who are judgmental.*” (IDI 8)

“*People would actually come and like call me names on “you terminated, you are such a bad person, how could you do that,” those sorts of judgments.*” (IDI 1).

A second co-existing, frequently reported belief was that pregnancy is unacceptable if the woman is young, unmarried, and/or from a poor socioeconomic background, due to the belief that the unborn child would be a financial burden to the community.

“*Pregnancy is also okay, but they think that you can’t be pregnant at that age, you know, a certain age. With pregnancy as well, the community is like this child does not prevent, or she does not use condoms.*” (IDI 9)

Similarly, pregnancy in young women was deemed inappropriate because of the perceived negative consequences for the women’s future opportunities.

“*The community, they are always judging, they say you are young, and you are not going to get anywhere [if you fall pregnant]. You don’t have a future.*” (IDI 4)

An inference that can be drawn is that there is an aspect of social injustice surrounding pregnancy choices among young women. In some such cases, community or family members seemed to encourage termination of the pregnancy.

“*In pregnancy, you are judged on what kind of family you come from, in my community. So, if my mom says don’t terminate, they [the community] were going to make sure that I terminate because they would say we are disgracing the community because my mom would be agreeing with teenage pregnancy and yet we are poor.*” (IDI 6)

“*I don’t know if my aunt means it, but she says if you fall pregnant, it is best to have an abortion* [termination].” (IDI 7)

Lastly, some participants also reported that their community beliefs around termination of pregnancy may be evolving and are divided, and some reported feeling supported by their community.

**“***At first people would down on your having terminated, but now we are able to talk about it and advise that you can actually terminate if you are not ready.*” (IDI 8)

“*Some see termination as a good decision, and some see it as a sin.*” (IDI 10)

“*I would say it is 50/50, you know, because around here there are mothers that are the same as mine, that are, they don’t believe in such things [termination], and then there are those that have the mind that it is her decision, there is nothing that you can do, and who are we, so yeah, my community is like this, it is 50/50.*” (IDI 5)

##### Pregnancy termination norms

The majority of the participants reported feeling that termination is common in their communities, despite the beliefs and stigma described above.

“*It’s common, but like if you are doing things on your own, since other people do it on their own, just like I did my one on my own, I am the only one who knew. So, I will say that it is common.” (IDI 9)*

“*I think it’s common because we hear hearsay and some, we see for ourselves, even though we don’t know if they terminated or if it was a miscarriage, we don’t know. So that happens in a lot of girls, it’s just that we are not sure.*” (IDI 5)

While acknowledging the secrecy around terminations of pregnancy, some participants found solace in the idea that termination of pregnancy is common, either because it allowed them to gain information from community members with previous experience, or because it lessened the perceived stigma around the decision.

“*It’s common because where I went, I know the place because I used to work there last of last year, and from that place … only one of my friends know about this, but most of my friends or people that I am familiar with, I know a lot of people who did termination. I feel like now that it is legal, it is not such a big issue like it was it first.*” (IDI 8)

“*Because they [members of her community] are not judgmental, and they accept the situation as it is. Yes, like with those that I know terminated before me, I asked for information from them wanting to know more.*” (IDI 10)

#### Media and policy

##### Social media portrayal and support around terminations

Although the portrayal of termination of pregnancy on social media was reported as a mixture of negative and supportive, some participants indicated receiving support from social media platforms, such as Facebook groups. This helped a number of participants make their decision.

*“Facebook, certain groups that I went to. Women talk about how they handled abortions* [termination]*. How it is okay to decide that you are going through termination.” (IDI 1)*

*“You know termination is not given that much time on social media but there is a group that I’m in on Facebook, there would be a child that posts saying that she is 16 and pregnant and she wants to terminate. There will be those who would try and convince her not to terminate and then there would be others who are harsh and say nasty things. So, I can say that there are those people who give support to others.” (IDI 2)*

*“It is talked about even on Facebook, that if you are not ready for a child, abort, you understand.” (IDI 8)*

## Discussion

In this study amongst young women in Soweto, South Africa, the reasons to terminate a pregnancy fell into the individual and interpersonal domains of the socioecological framework. Key reasons included financial dependence and insecurity, feeling unready to have a child (again), and a lack of support from family and partners. Additionally, we identified factors across all domains of the framework that, rather than being direct reasons for terminating the pregnancy, characterised the participants’ decision and their experience making the decision. The COVID-19 pandemic and resultant lockdown policies also indirectly impacted participants’ decisions through detrimental changes in financial situations and healthcare access.

The overall inference drawn from the findings of this study is that individuals exist within a context that is multi-layered and complicated. This complexity can be understood more clearly when recognising cross-cutting patterns for discussion, which include (lack of) sources of support, decision making within societal norms, structural socioeconomic and healthcare challenges, and feelings towards and experiences of the pregnancy. The relationship between the identified themes and these cross-cutting patterns has been illustrated in Supplementary Table 1.

Our findings showed that there tended to be a complex interplay between the reasons that led the young women to terminate their pregnancies, rather than one single reason. Results from previous studies, such as an exploration amongst Australian women, support this complex and “thoughtful” nature of reproductive decision making [24]. Previous quantitative and qualitative studies [24, 50, 51], including those from various African settings [12–14, 23, 29], similarly found that financial factors, feeling unready for motherhood, not wanting (more) children, the threat of losing educational and employment opportunities, and partner-relationship problems were important reasons for terminating a pregnancy. While the impact of family dynamics has arisen in previous research [24, 28], the emphasis tends to be on how partner factors influence pregnancy termination decision-making processes, as opposed to other family members. In this study, family aside from the partner, such as parents, siblings, cousins, and grandparents, may have played a more pronounced role in the decision to terminate given that many participants were unmarried, lived with their families and were financially dependent on their families.

Interestingly, in terms of decision making within societal norms, the participants in the current study overwhelmingly stated that the termination was their own decision. This was in contrast to a Kenyan study suggesting that male partners acted as principal decision makers for termination of pregnancy [28]. In the current study, participants expressed ownership and responsibility over the decision to terminate, even in the context of complex socioecological factors. This is more in line with findings from a US study which found that, while the nature of intimate partner relationships impacted women’s decisions, this did not tend to be through coercion, even amongst participants experiencing inter-partner violence [27]. In fact, according to participants in the current study, partners were sometimes not informed of the pregnancy/termination or seemed to remove themselves from the decision-making process. Since a lack of social-emotional support from family members and partners was also one of the key reasons to terminate, the independent nature of the women’s decision may also indicate a potential need for increased social-emotional support.

Our results highlighted a number of unmet needs around women’s access to reproductive health services in our setting. We identified a lack of health literacy around contraception and access to contraception services, which may contribute to unintended pregnancies in our setting [52, 53]. In addition, some of the participants used unsafe (illegal) termination services. Reasons for choosing this route reflect the ways in which the legal termination services in our setting remain inaccessible for women. For example, difficulty finding and getting to the clinics providing termination services, postponement of treatment by clinic staff, and fear of judgement from staff were some of the accessibility barriers for terminations at government clinics. The stigma around seeking and providing a pregnancy termination therefore impacts access to safe pregnancy termination services [54–56], despite the presence of legal termination services in South Africa. The lasting consequences this can have for women’s reproductive health is currently actively being highlighted in South African media [57].

In addition to gaps in healthcare services, our findings highlight the impact that structural factors and social influences have on the decision to terminate a pregnancy as well as the decision to use unsafe termination services. Such factors include, for example, (intergenerational) poverty, gaps in health literacy, and stigma from community and healthcare providers. This emphasises the need to engage multiple sectors when addressing unintended pregnancy and barriers to safe termination. While women in our study all reported that they thought termination of pregnancy was common in their communities, they also described experiencing multiple layers of stigma from these communities, including around poverty, pregnancy in unmarried women, and termination of pregnancy. These intersecting layers of stigma, and their influence on women’s experiences and reproductive decisions, highlights the importance of an intersectional approach when addressing stigma around pregnancy termination and barriers to safe pregnancy termination services [58].

A strength of this study was the use of the socioecological framework to explore reasons and influencing factors relevant to women’s decision to terminate, allowing for more in-depth insight into the context in which these decisions are made. One limitation of the study is that data was only collected from one study site (Soweto, South Africa). However, these findings align well with previous research, and the learnings from this study could give insight into other local and global settings with where women face similar challenges. Secondly, we were not able to explore how narratives differed between participants using safe (legal) vs unsafe termination services. This was outside of the scope of our research aim, and we felt that asking participants to declare this would not foster a safe environment for the interview. This could be an avenue for further research. In addition, the sensitive, stigmatised, and emotional nature of the study’s topic may have prevented participants from providing their full account to the interviewers. However, the interviews were carried out in an environment sensitive to the emotional nature of the topic, by a researcher who spoke the participants’ home language, and were experienced by the interviewers as notably forthright and in-depth.

In conclusion, in our study of young South African women, the decision to terminate pregnancy was placed in a socioecological framework, highlighting the complex structural and social context within which the decision is made. Unsafe termination of pregnancy remains a public health concern in South Africa [59, 60], and insight into the reasons why women choose to terminate, and the multifaceted, considered nature of the decision, can improve understanding of women’s lived experiences. This is necessary to better align legal termination services with women’s needs, for example by reducing judgement within healthcare settings and improving access to social and mental health support.

### Supplementary Information

**Additional file 1:** Semi-structured interview guide (.pdf).

**Additional file 2:** Supplementary Table 1. Oerview of the identified themes according to four cross-cutting points for discussion

## Data Availability

Data is available from the corresponding data upon reasonable request.

## Abbreviations

COVID-19: Coronavirus Disease 2019
HeLTI: Healthy Life Trajectories Initiative
IDI: In-depth interview
RDS: Respondent-driven sampling
