# Supplementary material for: A qualitative exploration of the reasons and influencing factors for pregnancy termination among young women in Soweto, South Africa: a Socio-ecological perspective
Source: Reprod Health. 2024 Jul 23;21:109. doi: 10.1186/s12978-024-01852-8 (PMC11265480; doi:10.1186/s12978-024-01852-8)
Supplement: Supplementary file 1 — Additional file 1: Semi-structured interview guide (.pdf). [file 12978_2024_1852_MOESM1_ESM.pdf]

Process evaluation of the *Bukhali* intervention trial

Interview guide – Termination of pregnancy

Directions: The qualitative in-depth interview begins with open-ended questions that point to starting a dialogue. The interviewer should encourage the study participant to do most of the talking but should use the questions listed here as a guide for discussion. After you ask each question, wait for the study participant to respond and only go on to the next question when you are satisfied with the answer. If it seems as though the study participant did not understand a question, then repeat it or ask it in another way until you are satisfied that they have understood. If the study participant goes on talking without much prompting, then let her guide the conversation. Mentally check off these questions as they are asked, or if they have been covered already through spontaneous discussion, so that you do not repeat a question if it has already been discussed previously.

Remember to audio record each interview. Use a backup recorder to prevent technological problems.

Preamble

1. Record the interview number, location, time of start and finish, participating audience, and your names as facilitator/s.
2. Thank the participants for showing up and welcome them to the meeting.
3. Discuss and set rules for the discussion, and ethical considerations.
    a. One person speaks at a time.
    b. There are no correct or wrong answers: be as transparent as possible in sharing your experience as it is, both the positive and the difficulties.
    c. Request to put phone on silent.
    d. Ask if there are other things people would like as guidelines for the discussion.
    e. While we'll ask most of the questions in English, feel free to respond in your language of choice if it makes it easier to express yourself.
4. Indicate that responses are confidential and anonymous.
5. Indicate the need of writing and recording.
6. Mention that it is expected that the interview will take 60 mins to complete
7. Ask participant to sign the consent to participate in the interview (if not already obtained).
8. Once obtained, test and start the recording.

Introduction

Thank you so much for coming today to speak about your experience of terminating your pregnancy. My name is Khuthala Mabetha and I am from Wits University in Johannesburg.

Proceed with questions for the relevant interview

Demographics and fertility history

1. What is your preferred language?
2. What is your age?
3. What is the highest level of education that you've attained?
4. Who do you live with at home?
5. Are you currently in a relationship?
    a. If yes, do you live with your partner?
    b. Are you married?
6. Do you have any children?
    a. If yes, how many children do you have?
    b. How many times have you been pregnant?
7. Probe (if necessary): Could you describe a normal day for you and your family?

With regards to your most recent pregnancy:

1. How did you feel about the pregnancy?
    a. Was it a planned pregnancy?
2. What were the circumstances surrounding your pregnancy?
3. How far were you in the pregnancy when you found out?
4. What influenced your decision to terminate the pregnancy?
5. What are the barriers that you experienced with regards to successfully terminating your pregnancy?
6. To what extent did these barriers affect your decision to terminate the pregnancy?
   OR
7. Are there any sources of motivation or barriers that influenced you to terminate your pregnancy?
8. Was this the first time you terminated a pregnancy?
    a. If yes, to what extent do you think that past experience influenced your decision and experience and in what way?

Intrapersonal

1. What are your personal beliefs about terminating a pregnancy?
    a. Do you hold any religious beliefs? Do you think those affect your beliefs about your pregnancy and termination?
    b. Do you think that pregnancy termination is common or uncommon in your community?
    c. To what extent do you think it is common or uncommon in your community to terminate a pregnancy?
2. How did these beliefs influence your decision to terminate the pregnancy?
3. How do you feel about having children in the future?

4. What are your views on contraception?
    a. Did you take contraception before you fell pregnant?
5. How easy is it for you to access contraception if you want it?
    a. Probe: geographic accessibility (close to a clinic), how young women are treated when asking for contraception.
6. How much power do you feel you have to make decisions about your reproductive health? (contraception use, sex life, negotiation of safe sex practices).
7. How did your work (or lack of work) or studies influence your decision to terminate the pregnancy?
8. To what extent do you feel it was your own decision to terminate the pregnancy?
9. Did you have someone to go to for information or questions when you fell pregnant? If so, where did the support come from?

Interpersonal factors

Family:

1. How would you describe your relationship with your family?
2. Did they know about your pregnancy and how did they feel about it?
    a. What are your family's beliefs about your pregnancy?
3. How did this affect your relationship with them?
4. How did they influence your decision to terminate the pregnancy?
5. Did they know about decision to terminate and how did they feel about it?
    a. What are your family's beliefs about terminating a pregnancy?
    b. How supported did you feel by your family in your decision to terminate the pregnancy?

Partner:

6. How would you describe your relationship with your partner?
7. Is your current partner the same as when you were pregnant?
8. How does he influence your decisions about contraception?
9. Did he know about your pregnancy and how did he feel about it?
    a. What are your partner's beliefs about your pregnancy?
10. How did this affect your relationship with him?
11. How did he influence your decision to terminate the pregnancy?
12. Did he know about decision to terminate and how did he feel about it?
    a. What are your partner's beliefs about terminating a pregnancy?
    b. How supported did you feel by your partner in your decision to terminate the pregnancy?

Peers:

1. Did you have a friend or peer who supported you in your decision to terminate the pregnancy? If so, in what way did they support you?
2. To what extent was your decision to terminate your pregnancy impacted by peers or a friend?
3. How did this affect your relationship with them?
4. How supported did you feel by a friend or friends in your decision to terminate the pregnancy?
5. Did a peer or friend influence your decision about contraception?

Institutions and Organizations / Community

1. What organizations supported you in your decision to terminate the pregnancy? (Health advocates, schools, clinics, youth clinics).
2. What is your religious affiliation?
    a. What are your church's values around pregnancy and termination?
    b. How did these values influence your decision?
3. How did your level of access to and support from your clinic influence your decision to terminate the pregnancy?
4. What were the clinic staff attitudes toward your pregnancy?
    a. To what extent did these attitudes influence your decision to terminate your pregnancy?
5. What are your community's beliefs around pregnancy and termination?
6. How safe do you feel in your neighbourhood?
    a. How do you think that impacted your decision to terminate the pregnancy?

Public policy

1. What are your views on how media portrays pregnancy and termination?
    a. Is there anything on media pertaining to pregnancy (TV, Facebook, twitter, radio, advertising) that influenced your decision to terminate the pregnancy?
2. How easy or difficult was it to access services to terminate your pregnancy? Why?

COVID-19

1. How do you think COVID-19 impacted your pregnancy and termination? In what way? (socioeconomic circumstances, fear of going to the clinic, lockdown regulations)

Additional reasons

1. Are there any other reasons or factors that contributed to your decision to terminate your pregnancy?

Mental health

1. How would you describe the impact that terminating your pregnancy has had on your emotional well-being (mental health)?
    a. If negative impact: What support have you had to deal with this?
2. Thinking about the termination of your pregnancy, is there anything you would have done differently or would do differently in the future, if you fell pregnant again?
3. Is there anything else you would like to share about this experience that we haven't covered?
