# Supplementary material for: A qualitative exploration of the reasons and influencing factors for pregnancy termination among young women in Soweto, South Africa: a Socio-ecological perspective
Source: Reprod Health. 2024 Jul 23;21:109. doi: 10.1186/s12978-024-01852-8 (PMC11265480; doi:10.1186/s12978-024-01852-8)
Supplement: Supplementary file 2 — Additional file 2: Supplementary Table 1. Oerview of the identified themes according to four cross-cutting points for discussion [file 12978_2024_1852_MOESM2_ESM.docx]

| **Cross-cutting**  **patterns**  **Domains** | **Feelings towards and experience of pregnancy** | **Decision making within societal norms and expectations** | **Lack of vs sources of support** | **Structural socioeconomic and healthcare challenges** |
| --- | --- | --- | --- | --- |
| **Individual-level** | **Lack of readiness and desire for pregnancy and motherhood**  **Pregnancy related mental and physical conditions** | **Pregnancy related mental and physical conditions.**  Sense of agency over termination  Personal beliefs around termination |  | **Financial insecurity and dependence**  **Impact on employment and educational opportunities** |
| **Interpersonal** |  | Role of partner in decision to terminate | **Lack of support or stability from partner**  **Lack of family support**  **Threat of an adverse impact on family dynamics**  Social support from confidants |  |
| **Institutions and organisations** |  |  | Organisations and institutions that offer support | Accessibility of termination at government clinics, private clinics, and illegal termination services |
| **Community** |  | Community socio-cultural beliefs around termination and pregnancy  Pregnancy termination norms |  |  |
| **Media and policy** |  |  | Social media portrayal of termination |  |

*Bold = reasons (rather than factors characterizing the decision)
